# Effect of health education based on the Transtheoretical Model on pregnant women’s knowledge, attitudes, and practices toward painless delivery: a quasi-experimental study

**DOI:** 10.3389/fpubh.2026.1763939

**Published:** 2026-09-17

**Authors:** Yu Yang, Yuanyuan Wu, Zemei Mao, Li Wang

**Affiliations:** 1 Department of Gynecology and Obstetrics, Wuhan Children’s Hospital (Wuhan Maternal and Child Healthcare Hospital), Tongji Medical College, Huazhong University of Science & Technology, Wuhan, China; 2 Department of Anesthesiology, Wuhan Children’s Hospital (Wuhan Maternal and Child Healthcare Hospital), Tongji Medical College, Huazhong University of Science & Technology, Wuhan, China

**Keywords:** attitudes, health education, knowledge, labor, obstetric, pain, pregnant women, Transtheoretical Model

## Abstract

**Background:**

Traditional methods have limited reach and sustainability and often fail to increase the actual uptake of epidural analgesia. This study explored the impact of health education grounded in the Transtheoretical Model (TTM) on primiparous women’s knowledge, attitudes, and practices (KAP) regarding painless delivery, as well as associated delivery outcomes.

**Materials and methods:**

This quasi-experimental study was conducted at Wuhan Children’s Hospital from December 2024 to April 2025. Eligible primiparous women planning vaginal delivery were assigned to the TTM-based intervention group or the routine education group according to enrollment sequence. All participants received epidural analgesia during labor. Baseline and post-intervention assessments measured KAP using a self-developed questionnaire, and childbirth experience using the Chinese version of the Childbirth Experience Questionnaire (CEQ-C).

**Results:**

A total of 148 women were enrolled (TTM group: *n* = 77; routine education group: *n* = 71). All participants received epidural analgesia during labor as part of routine clinical practice. Post-intervention, the TTM group showed greater improvements in KAP scores (all *p* < 0.001) compared with the routine education group. At follow-up, KAP scores remained higher in the TTM group (all *p* < 0.001). CEQ-C scores did not differ significantly between groups (2.74 vs. 2.63, *p* = 0.062). Multivariable linear regression analysis indicated occupational role (*β* = −0.113, *p* = 0.014) and delivery satisfaction (*p* < 0.001) as independent predictors of CEQ-C scores.

**Conclusion:**

TTM-based individualized education significantly improves KAP levels in primiparous women. These findings suggest that implementing TTM interventions in clinical practice can optimize perinatal education.

## Introduction

Labor pain is almost universal among parturients and is widely recognized as one of the most severe forms of pain in a woman’s lifetime (1). Beyond causing intense physical discomfort, inadequately managed labor pain has been associated with adverse psychological outcomes, including postpartum depression, as well as physiological stress responses that may compromise maternal-fetal outcomes (2, 3). Epidural analgesia, commonly referred to as painless delivery, is considered the gold standard for labor pain relief (4). In high-income countries, more than 70% of women use epidural analgesia, whereas in China, awareness and uptake are substantially lower, with approximately 64% of women aware of this option and only about 30% receiving it (5). This discrepancy suggests persistent gaps in knowledge, attitudes, and practices regarding painless delivery (5).

Knowledge-attitude-practice (KAP) surveys are structured instruments used to evaluate what individuals know about a health issue, how they feel about it, and how they behave in relation to it (6). By revealing knowledge gaps and misconceptions, KAP studies provide an evidence base for developing targeted health education interventions (7). In maternal health research, KAP surveys have been widely applied among pregnant women. For example, a recent multicenter study in Palestine reported that although 97.9% of pregnant women were aware of epidural analgesia, only 62.5% stated they would choose it if cost were not a barrier (8). These findings indicate that, despite generally positive attitudes toward painless delivery, women’s knowledge remains limited, underscoring the need for focused antenatal education. Evidence further shows that targeted educational interventions can significantly enhance women’s knowledge and willingness to use labor analgesia (8, 9). However, traditional approaches such as brief counseling or distribution of pamphlets have limited reach and sustainability and often do not translate into increased actual use of epidural analgesia (10).

The Transtheoretical Model (TTM) offers a promising framework to overcome these limitations. It conceptualizes health behavior change as a dynamic process that progresses through five stages (precontemplation, contemplation, preparation, action, and maintenance), thereby supporting the design of tailored, stage-specific interventions (11, 12). TTM-based strategies have demonstrated effectiveness in promoting various health behavior changes (13). In the context of pregnancy, TTM-guided interventions have been increasingly used to support health-promoting behaviors, including reducing alcohol-exposed pregnancies (14, 15), improving oral hygiene and dietary habits (16), and facilitating weight management and prenatal care through structured counseling (17). Yet, research on painless childbirth has predominantly focused on clinical practice and safety, with relatively few systematic educational interventions aimed at improving pregnant women’s KAP and psychological wellbeing (18).

To address this gap, the present study evaluates the effects of a TTM-based health education program on pregnant women’s KAP regarding painless delivery and examines its impact on delivery quality and psychological health. By comparing outcomes between women receiving stage-specific TTM-guided education and those receiving conventional education, this quasi-experimental study seeks to provide empirical evidence on the effectiveness of TTM-based health education in enhancing awareness, fostering positive attitudes, supporting informed practices, and ultimately improving childbirth experiences and maternal wellbeing.

## Materials and methods

This study is reported following the TREND statement (Improving the reporting quality of nonrandomized evaluations of behavioral and public health interventions) (19). The TREND checklist has been provided as Supplementary materials.

### Study design and participants

A quasi-experimental study was conducted at Wuhan Children’s Hospital (Wuhan Women and Children Medical Center), an affiliated hospital of Tongji Medical College, Huazhong University of Science and Technology, between December 2024 and April 2025. The study protocol received institutional ethics committee approval (#2024R143-E01), and written informed consent was obtained from all participants. The trial was prospectively registered with the Chinese Clinical Trial Registry (ChiCTR; #ChiCTR2500112193) and the National Health Information Platform/Medical Research Registration and Filing System (#MR-42-26-013444), with ethical approval and registration completed prior to patient enrollment.

Eligible participants were women with uncomplicated pregnancies who were planning vaginal delivery, had no serious complications or contraindications to neuraxial labor analgesia, and possessed adequate language, auditory, and cognitive abilities to understand and complete the intervention. Women scheduled for cesarean section due to severe obstetric complications, those with a history of major psychiatric or neurological disorders, or those unable to provide informed consent were excluded. All enrolled participants received epidural analgesia during labor. Women were withdrawn from the study if they delivered preterm (<37 weeks), were unable to receive neuraxial analgesia because of emergencies, contraindications, or technical failure, or did not complete at least 6 weeks of health education or follow-up.

At this center, neuraxial labor analgesia (primarily epidural analgesia) is routinely offered as a standard option for medically eligible primiparous women planning vaginal delivery, but it is not mandatory. Each woman decided to receive or decline neuraxial analgesia after routine counseling by the obstetric and anesthesiology teams. In this study, women were eligible regardless of their initial analgesia preference; those who ultimately did not receive neuraxial analgesia due to emergency circumstances, contraindications, technical failure, preterm birth, or conversion to cesarean delivery were excluded from the final per-protocol analysis because they lacked complete outcome data.

This TTM-based health education program was delivered during the antenatal period and completed before the onset of labor. All components of the intervention, including in-person counseling and remote follow-up, were implemented prior to admission for delivery.

### Questionnaire

In line with the study objectives, and drawing on the Expert Consensus on Neuraxial Labor Analgesia in China (2021 edition) (20), a KAP questionnaire on labor analgesia was developed for pregnant women (21). The instrument consisted of four sections. The first section collected demographic and baseline information, including maternal age, gestational age, body mass index, type of medical insurance, place of residence, marital status, highest educational level, employment status, household income, pre-pregnancy medical history, and the spouse’s education and employment status. The second section assessed knowledge of labor analgesia using 10 items plus one “trap” item, and the third section evaluated attitudes and beliefs regarding labor analgesia using 11 items. The fourth section captured health-related behaviors in daily life through five items.

Scoring methods were tailored to each dimension. Knowledge items were rated from “very familiar” (2 points) to “unclear” (0 points), yielding a total score from 0 to 18. Attitudes were measured on a five-point Likert scale from “very positive” (5) to “very negative” (1), with total scores ranging from 10 to 50. Practices were evaluated using the same five-point Likert scale, yielding total scores ranging from 5 to 25. In this context, because neuraxial labor analgesia is routinely offered to eligible primiparous women and all analyzed participants ultimately received epidural analgesia, the “practice” dimension primarily captured preparatory and engagement behaviors (e.g., seeking information, attending antenatal labor analgesia evaluations, participating in online consultations, and planning for analgesia use) rather than the binary decision to accept or decline neuraxial analgesia.

In a pilot test involving 30 valid questionnaires, the Cronbach’s *α* coefficient was 0.863, indicating good internal consistency and reliability of the instrument.

### Intervention measures

Participants were allocated to groups based on the parity of their enrollment sequence number: odd numbers to the intervention group and even numbers to the control group. The TTM-based health education program aimed to provide balanced, evidence-based information on the benefits, limitations, and risks of neuraxial labor analgesia and to correct common misconceptions. The intervention was explicitly framed as support for informed decision-making rather than promotion of a specific mode of pain relief. Throughout antenatal care and during labor, women retained full autonomy to accept or decline neuraxial analgesia, and choices for non-pharmacological or “natural” childbirth were respected in clinical practice.

The intervention group received a health education program designed according to the TTM (Supplementary Table S1) and informed by baseline KAP survey findings. The program was structured around the five TTM stages of behavior change (precontemplation, contemplation, preparation, action, and maintenance) and combined information provision, motivation enhancement, skills training, and social support, with the primary aim of strengthening pregnant women’s KAP regarding labor analgesia.

At enrollment, anesthesiology and obstetric staff used a brief, standardized set of questions to assess women’s awareness of neuraxial labor analgesia, perceived risks, and current intentions, and to classify their motivational stage within the TTM framework. In this setting, where baseline familiarity with epidural analgesia is generally low, most participants fell along a continuum between precontemplation (limited awareness and defensive or avoidant attitudes) and contemplation (emerging awareness with ambivalence), and clinical judgment was used to differentiate these patterns rather than a single quantitative scoring tool. For women closer to precontemplation, initial contacts focused on “consciousness-raising,” including clarification of basic concepts and correction of common misconceptions, whereas for those in contemplation, counseling emphasized balanced discussion of benefits and risks and support for informed decision-making. As women demonstrated readiness to plan for neuraxial analgesia, the intervention advanced to preparation and action components, such as individualized counseling in a dedicated labor analgesia clinic, development of personalized prenatal health plans, and practical planning for analgesia use. In later pregnancy, structured one-to-one follow-up (via telephone and online messaging) and a dedicated follow-up group were used to reinforce decisions and address emerging concerns, consistent with the maintenance stage. Educators (obstetric and anesthesiology staff) completed standardized training in TTM-based counseling, KAP assessment, and use of stage-specific materials (brochures, videos, and messaging templates), and received ongoing supervision to ensure fidelity to stage-matched, individualized delivery.

A TTM communication team was established with clearly defined roles for the leader, deputy, and members. All team members underwent standardized training in TTM theory, KAP methodology, and relevant health education content, and only those who passed competency assessments were authorized to deliver the intervention. During antenatal visits and follow-up contacts, all enrolled women were invited to involve key family members (most often spouses or primary caregivers) in educational activities. Family members were encouraged, but not required, to attend lectures, participate in one-to-one counseling sessions when present, and join group discussions. Educational brochures and videos were routinely provided to both women and available family members, and the same online messaging channels used for participant follow-up were open to family inquiries. Thus, all families had equal opportunity to receive information and support; the extent of actual participation depended on their availability and willingness to engage. Group discussions were organized to identify potential barriers and explore coping strategies. Family members were also educated, based on KAP findings, to improve their understanding and attitudes toward labor analgesia and to strengthen their support for women during pregnancy and childbirth. The invitation to involve family members and access family-oriented educational materials was systematically extended to all participants in the intervention group, and no family was excluded based on sociodemographic or clinical characteristics. The intervention lasted at least 6 weeks. The control group received routine hospital-based education on painless delivery, without individualized or stage-specific components.

### Evaluation indicators

The evaluation indicators included a self-developed KAP questionnaire on labor analgesia, the Self-Rating Depression Scale (SDS), the Chinese version of the Childbirth Experience Questionnaire (CEQ-C), pain scores, and delivery outcomes. Before the intervention, both groups completed baseline assessments capturing demographic and clinical data, including age, gestational week, body mass index, type of medical insurance, place of residence, marital status, educational level, employment status, household income, pre-pregnancy medical history, and the spouse’s education and employment status. The KAP questionnaire was used to assess women’s baseline knowledge, attitudes, and practices regarding labor analgesia. Psychological status was evaluated using the SDS, a 20-item scale rated on four response options; raw scores were multiplied by 1.25 and rounded to the nearest integer to obtain standardized scores, with higher scores indicating more severe depressive tendencies (22).

Six weeks after the intervention, participants were reassessed with the same instruments to examine changes in KAP levels and psychological status, and to analyze between-group differences. Childbirth experiences were additionally assessed within 2 weeks postpartum using the CEQ-C, which comprises 19 items across four domains (sense of self-control, professional support, personal perception, and participation), each rated on a four-point Likert scale (23). Negatively worded items were reverse-scored, and overall and domain-specific scores were calculated as mean values, with higher scores reflecting a more positive childbirth experience. Delivery-related outcomes were also recorded, including pain scores before and after labor analgesia, persistence of pain within 24 h postpartum, satisfaction with analgesia, mode of delivery (spontaneous vaginal, emergency cesarean after attempted vaginal delivery, or forceps-assisted), postpartum blood loss, and complications related to labor analgesia (such as lower limb numbness or weakness, pruritus, headache, or prolonged second stage of labor). Neonatal disposition following birth, including return to the obstetric ward or referral to the neonatology department, was also documented.

Delivery satisfaction was assessed separately using a single global categorical item (dissatisfied, fair, satisfied, or very satisfied). This item was not part of the CEQ-C and was scored independently; however, both measures reflect women’s subjective appraisal of childbirth and may capture related constructs.

### Statistical analysis

Data were analyzed using SPSS version 27.0 (IBM, Armonk, NY, USA). Continuous variables with a normal or approximately normal distribution are presented as mean ± standard deviation (SD), and non-normally distributed variables as median with interquartile range (IQR). Categorical variables are summarized as frequencies and percentages. Between-group differences were examined using the Mann–Whitney *U* test for continuous variables and the chi-square test or Fisher’s exact test for categorical variables. Within-group pre-post changes in KAP scores and psychological status (SDS) were evaluated using the Wilcoxon signed-rank test, as difference scores did not meet the assumptions of normality. Post-intervention comparisons between the TTM group and the routine education group were performed using the Mann–Whitney *U* test for KAP levels, psychological outcomes, and childbirth experience scores derived from the Chinese version of the CEQ-C. Multivariable linear regression was also used to examine the influence of demographic variables on the childbirth experience.

The three prespecified primary outcomes were the KAP dimension scores. For these outcomes, a Bonferroni correction was applied to account for the three primary comparisons, yielding an adjusted two-sided significance threshold of *p* < 0.0167 (0.05/3). *p* values reported in the tables and text are raw, unadjusted *p* values; statistical significance for the primary KAP outcomes was determined using the adjusted threshold. SDS, CEQ-C, and delivery-related outcomes were treated as secondary or exploratory outcomes and were assessed using the conventional two-sided threshold of *p* < 0.05, without formal adjustment for multiple comparisons. These secondary and exploratory findings should therefore be interpreted cautiously because of the increased risk of type I error.

All analyses were conducted on a per-protocol basis and included only participants who completed the full intervention and follow-up assessments. Women were excluded from the final analysis if they delivered preterm (less than 37 weeks), were unable to receive neuraxial analgesia due to emergency circumstances, contraindications, or technical failure, or did not complete at least 6 weeks of health education or follow-up. Because excluded participants did not have post-intervention outcome data (primarily due to conversion to cesarean delivery or preterm birth), intention-to-treat analysis with imputation was not feasible. To assess potential attrition bias, baseline characteristics of completers and excluded participants were compared (Supplementary Table S3).

The planned sample size of 200 primiparous women (100 per group) was determined pragmatically, based on the expected number of eligible patients during the recruitment period and the available resources. No formal *a priori* sample-size or power calculation was performed. Therefore, the study may have had limited ability to detect modest between-group differences, particularly for secondary outcomes such as childbirth experience.

## Results

### Participant flow

**Figure 1:**
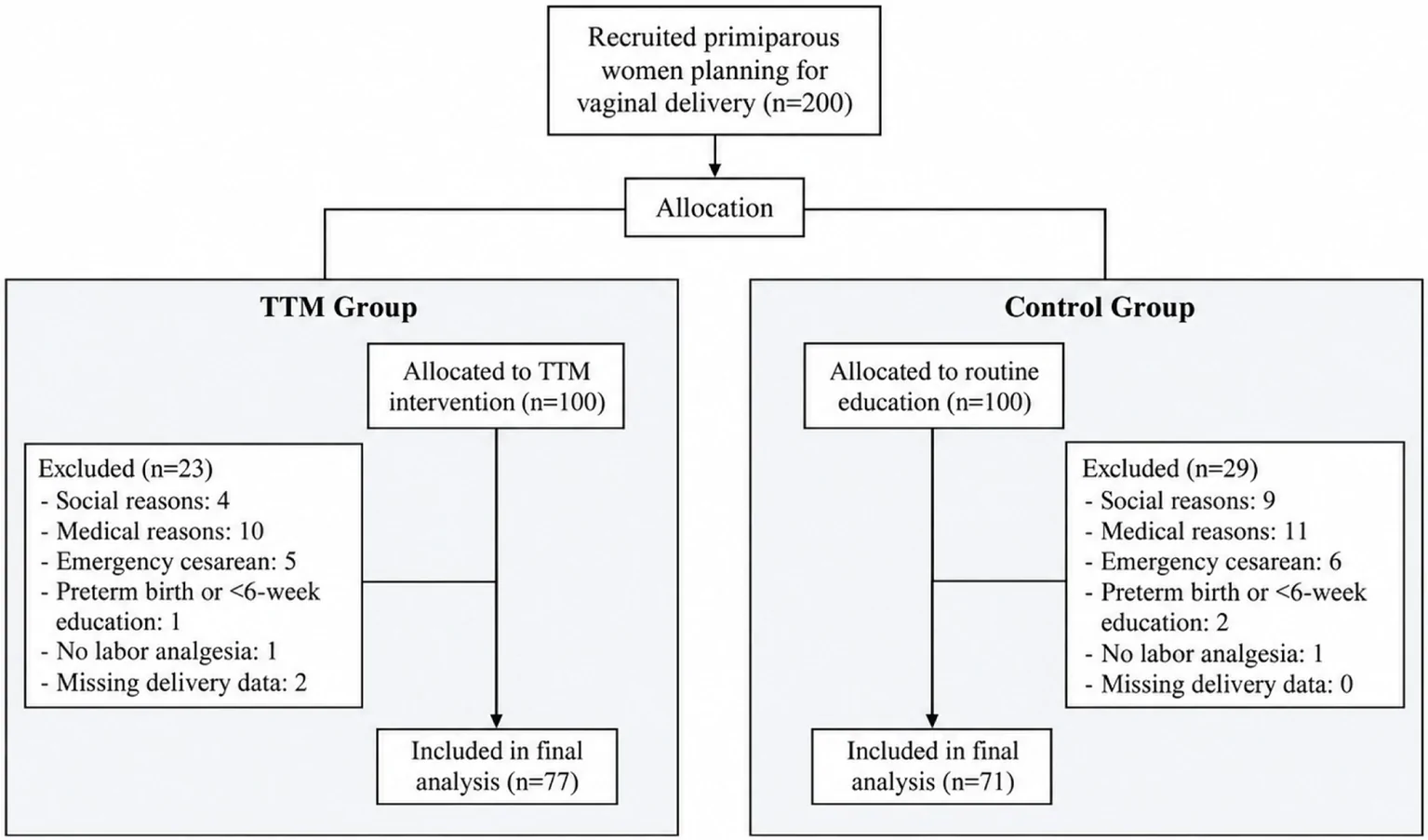
Participant flowchart: enrollment of pregnant women, allocation to the TTM-based intervention group or the routine education group, and inclusion in the final per-protocol analysis.

A total of 200 primiparous women without serious pregnancy complications who were planning vaginal delivery were enrolled, with 100 allocated to the TTM group and 100 to the routine education group. After applying the exclusion criteria, 23 women in the TTM group and 29 in the control group were excluded. The most frequent reasons for exclusion were cesarean delivery for medical indications (*n* = 21, 40.4%), elective cesarean delivery for social reasons (*n* = 13, 25.0%), and emergency cesarean section after an attempted vaginal delivery (*n* = 11, 21.2%). Other reasons included preterm delivery (*n* = 3), inability to receive neuraxial analgesia (*n* = 2), and incomplete follow-up data (*n* = 2; Supplementary Table S2). Ultimately, 77 women in the TTM group and 71 in the control group were included in the final analysis (Figure 1). Overall, 52 of the 200 initially enrolled women were excluded, corresponding to an attrition rate of approximately 26% (23% in the TTM group and 29% in the routine education group). A comparison of baseline demographic and clinical characteristics between completers and excluded participants showed no statistically significant differences in age, residence, education, occupation, monthly income, or type of medical insurance (Supplementary Table S3).

### Baseline characteristics

A total of 148 primiparous women were included in the analysis (TTM group: *n* = 77; routine education group: *n* = 71). The two groups were broadly comparable in terms of demographic, clinical, and obstetric characteristics. However, women in the TTM group demonstrated markedly greater engagement with perinatal education resources. Delivery satisfaction was also higher in the TTM group, with a larger proportion of women reporting being very satisfied (Table 1).

**Table 1:** Basic and clinical characteristics.

Variables	Total (*N* = 148)	TTM group (*N* = 77)	Routine education group (*N* = 71)	*p*
		Median (P25, P75)/*n* (%)	Median (P25, P75)/*n* (%)	
Age (years)	29 (27.25, 32)	29 (27, 32)	29 (28, 32)	0.902
Residence				0.903
Urban	107 (72.30%)	56 (72.73%)	51 (71.83%)	
Suburban/rural	41 (27.70%)	21 (27.27%)	20 (28.17%)	
Education				0.597
High school/technical secondary school or below	18 (12.16%)	8 (10.39%)	10 (14.08%)	
Associate degree	37 (25.00%)	19 (24.68%)	18 (25.35%)	
Bachelor’s degree	64 (43.24%)	37 (48.05%)	27 (38.03%)	
Master’s degree or above	29 (19.59%)	13 (16.88%)	16 (22.54%)	
Current employment status				0.866
Management/professional and technical personnel	44 (29.73%)	24 (31.17%)	20 (28.17%)	
General staff/commercial service workers	63 (42.57%)	33 (42.86%)	30 (42.25%)	
Other laborers	41 (27.70%)	20 (25.97%)	21 (29.58%)	
Monthly household income (RMB)				0.498
2,000–5,000	34 (22.97%)	16 (20.78%)	18 (25.35%)	
5,000–10,000	59 (39.86%)	29 (37.66%)	30 (42.25%)	
10,000–20,000	39 (26.35%)	21 (27.27%)	18 (25.35%)	
>20,000	16 (10.81%)	11 (14.29%)	5 (7.04%)	
Are you planning to choose painless childbirth for this delivery?				0.690
Yes	104 (70.27%)	53 (68.83%)	51 (71.83%)	
Depends on the situation	44 (29.73%)	24 (31.17%)	20 (28.17%)	
Type of medical insurance				0.939
Only social medical insurance	75 (50.68%)	38 (49.35%)	37 (52.11%)	
Both social and commercial medical insurance	52 (35.14%)	28 (36.36%)	24 (33.80%)	
Others	21 (14.19%)	11 (14.29%)	10 (14.08%)	
Number of prenatal check-ups	12 (11, 13.75)	12 (11, 14)	12 (10, 13)	0.052
Gestational week at delivery				0.555
37 to 37+	14 (9.46%)	8 (10.39%)	6 (8.45%)	
38 to 38+	35 (23.65%)	18 (23.38%)	17 (23.94%)	
39 to 39+	78 (52.70%)	43 (55.84%)	35 (49.30%)	
40 to 40+	21 (14.19%)	8 (10.39%)	13 (18.31%)	
Total labor duration (min)	505 (390, 701.25)	470 (390, 697.5)	560 (385, 710)	0.387
Total analgesia duration (min)	385 (281.25, 517.5)	365 (280, 515)	425 (285, 520)	0.307
Analgesia time/total labor duration ratio	0.75 (0.68, 0.8)	0.75 (0.68, 0.8)	0.75 (0.68, 0.81)	0.980
Mode of delivery				0.921
Cesarean	84 (56.76%)	44 (57.14%)	40 (56.34%)	
Vaginal	64 (43.24%)	33 (42.86%)	31 (43.66%)	
Postpartum blood loss (ml)	300 (230, 400)	300 (220, 400)	300 (230, 410)	0.665
Pain score (NRS) before analgesia	7 (7, 8)	7 (7, 8)	7 (7, 8)	0.910
Pain score (NRS) 20 min after analgesia	3 (2, 3)	3 (2, 3)	3 (2, 3)	0.982
Pain score (NRS) at full cervical dilation	6 (6, 7)	6 (6, 7)	6 (5, 7)	0.724
Delivery satisfaction				0.017
Dissatisfied	12 (8.11%)	3 (3.90%)	9 (12.68%)	
Fair	22 (14.86%)	8 (10.39%)	14 (19.72%)	
Satisfied	58 (39.19%)	29 (37.66%)	29 (40.85%)	
Very satisfied	56 (37.84%)	37 (48.05%)	19 (26.76%)	
Safety indicators of painless delivery				
Puncture failure				0.427
Yes	6 (4.05%)	2 (2.60%)	4 (5.63%)	
No	142 (95.95%)	75 (97.40%)	67 (94.37%)	
Headache				0.350
Yes	4 (2.70%)	1 (1.30%)	3 (4.23%)	
No	144 (97.30%)	76 (98.70%)	68 (95.77%)	
Nerve injury				
No	148 (100.00%)			
Intrapartum temperature ≥ 38 °C				0.891
Yes	13 (8.78%)	7 (9.09%)	6 (8.45%)	
No	135 (91.22%)	70 (90.91%)	65 (91.55%)	
Prolonged second stage of labor				
No	148 (100%)			
Neonatal Apgar score: 1 min	9 (9, 9)	9 (9, 9)	9 (9, 9)	0.609
Neonatal Apgar score: 5 min	10 (10, 10)	10 (10, 10)	10 (10, 10)	0.649

### Changes in KAP and psychological status before and after intervention

After the intervention, KAP scores improved substantially, with larger gains in the TTM group than in the routine education group. Across the full sample, median knowledge, attitude, and practice scores increased. Within-group analysis showed pronounced improvements in the TTM group, while the routine education group showed more modest but still significant gains (Table 2).

**Table 2:** Changes in participants’ KAP levels and psychological status before and after the intervention.

Outcome measure	Before intervention	After intervention	*p*
Knowledge	6 (4, 10)	15 (11, 17)	<0.001
Attitude	44 (41, 50.75)	47 (44, 52)	<0.001
Practice	11 (9, 15)	15 (13, 18)	<0.001
SDS	39.5 (33.25, 45.75)	42 (38.25, 44)	0.139
TTM group			
Knowledge	6 (4, 9.5)	17 (15.5, 18)	<0.001
Attitude	45 (41, 51)	49 (45.5, 54)	<0.001
Practice	11 (9, 15)	16 (15, 18)	<0.001
SDS	39 (33, 47)	42 (38, 45)	0.361
Routine education group			
Knowledge	7 (3, 10)	11 (8, 14)	<0.001
Attitude	44 (42, 48)	45 (43, 50)	0.099
Practice	12 (9, 15)	14 (12, 17)	0.003
SDS	40 (34, 45)	41 (39, 44)	0.058

Values are presented as median (P25, P75).

The possible score ranges are Knowledge: 0, 18; Attitude: 10, 50; Practice: 5, 25; SDS: 20, 100.

*p* values are raw, unadjusted values. For the prespecified primary outcomes (KAP dimension scores), statistical significance was assessed using the Bonferroni-adjusted threshold of *p* < 0.0167. SDS and CEQ-C were secondary outcomes and were evaluated using *p* < 0.05.

### Post-intervention comparison between groups

At the end of the intervention, women in the TTM group had higher scores across all three KAP dimensions than those in the routine education group. They had more antenatal labor analgesia evaluations and more online consultations than those in the routine education group. SDS scores were similar between groups. Childbirth experience scores (CEQ-C) also did not differ significantly (Table 3). Although CEQ-C scores were slightly higher in the TTM group, this difference did not reach statistical significance. In multivariable analysis, higher global delivery-satisfaction ratings and occupational role were associated with CEQ-C scores. Because global delivery satisfaction and CEQ-C both reflect subjective evaluations of childbirth, this association should not be interpreted as demonstrating an independent causal effect of satisfaction on childbirth experience. Rather, it likely reflects the close relationship between women’s overall satisfaction with delivery and their multidimensional appraisal of childbirth. These findings suggest that intrapartum care processes and women’s expectations may have had a stronger influence on perceived childbirth experience than antenatal education alone.

**Table 3:** Comparison of intervention-related process indicators and intervention effects between the two groups.

Indicator	TTM group	Routine education group	*p*
Process indicators			
Number of antenatal painless delivery evaluations	1 (1,1)	0 (0,1)	<0.001
Number of online consultations	3 (2,4)	1 (0,2)	<0.001
Post-intervention outcomes			
Knowledge	17 (15.5, 18)	11 (8, 14)	<0.001
Attitude	49 (45.5, 54)	45 (43, 50)	<0.001
Practice	16 (15, 18)	14 (12, 17)	<0.001
SDS	42 (38, 45)	41 (39, 44)	0.817
CEQ-C	2.74 (2.53, 2.84)	2.63 (2.42, 2.79)	0.062

Values are presented as median (P25, P75).

The possible score ranges are Knowledge: 0, 18; Attitude: 10, 50; Practice: 5, 25; SDS: 20, 100; CEQ-C: 1, 4.

*p* values are raw, unadjusted values. For the prespecified primary outcomes (KAP dimension scores), statistical significance was assessed using the Bonferroni-adjusted threshold of *p* < 0.0167. SDS and CEQ-C were secondary outcomes and were evaluated using *p* < 0.05.

Overall, both groups showed improvement over time, but TTM-based education led to larger and more consistent gains across all KAP domains than routine education. The higher post-intervention practice scores observed in the TTM group, together with their greater number of antenatal labor analgesia evaluations and online consultations, suggest more active information-seeking, greater engagement with counseling, and more proactive planning for neuraxial analgesia. This pattern supports the hypothesis that stage-tailored, repeatedly reinforced education can enhance behavioral readiness and promote more frequent pain-related self-management behaviors.

### Exploratory multivariable linear regression analysis

Multivariable linear regression showed that occupational role and delivery satisfaction were independently associated with CEQ-C scores (Table 4).

**Table 4:** Exploratory multivariable linear regression analysis of factors associated with CEQ-C score.

Variable	*β*	95% CI	*p*
Intervention method			
TTM	−0.05	−0.125, 0.026	0.199
Routine education	ref		
Age	0.008	−0.001, 0.018	0.079
Residence			
Urban	−0.032	−0.106, 0.042	0.393
Suburban/rural	ref		
Education			
High school/technical secondary school or below	ref		
Associate degree	0.085	−0.025, 0.195	0.127
Bachelor’s degree	0.099	−0.019, 0.217	0.101
Master’s degree or above	0.073	−0.067, 0.212	0.303
Current employment status			
Management/professional and technical personnel	−0.113	−0.203, −0.023	**0.014**
General staff/commercial service workers	−0.05	−0.123, 0.023	0.178
Other laborers	ref		
Monthly household income (RMB)			
2,000–5,000	ref		
5,000–10,000	−0.031	−0.121, 0.059	0.494
10,000–20,000	0.047	−0.059, 0.153	0.378
>20,000	0.039	−0.091, 0.169	0.552
Type of medical insurance			
Only social medical insurance	−0.005	−0.105, 0.095	0.925
Both social and commercial medical insurance	−0.018	−0.137, 0.101	0.763
Others	ref		
Number of antenatal painless delivery evaluations	−0.011	−0.07, 0.048	0.72
Number of online consultations	0.001	−0.023, 0.026	0.912
Number of prenatal check-ups	−0.007	−0.023, 0.009	0.375
Gestational week at delivery			
37 to 37+	ref		
38 to 38+	0.019	−0.091, 0.13	0.729
39 to 39+	0.001	−0.103, 0.104	0.996
40 to 40+	0.035	−0.097, 0.167	0.6
Mode of delivery			
1	ref		
2	0.005	−0.055, 0.064	0.879
Pain score (NRS) before analgesia	−0.004	−0.036, 0.029	0.821
Pain score (NRS) 20 min after analgesia	0.008	−0.03, 0.046	0.668
Pain score (NRS) at full cervical dilation	0.003	−0.029, 0.035	0.851
Delivery satisfaction			
Dissatisfied	ref		
Fair	0.345	0.216, 0.474	**<0.001**
Satisfied	0.76	0.627, 0.893	**<0.001**
Very satisfied	0.9	0.748, 1.053	**<0.001**

Model fit: *R*^2^ = 0.784; adjusted *R*^2^ = 0.757; *F* = 29.660; *p* < 0.001. Bold values indicate statistically significant associations (*p* < 0.05).

## Discussion

TTM-based health education improved primiparous women’s knowledge, attitudes, and practices regarding painless delivery and enhanced their overall preparedness for childbirth.

The stage-based behavioral intervention grounded in the TTM led to clear gains in knowledge, attitudinal orientation, and self-reported preparedness for labor analgesia, especially in areas requiring active decision-making. Participants in the intervention group were more likely to seek consultations and ask questions about pain management options, aligning with previous reports of behaviorally stratified interventions in maternal health (24).

However, changes in beliefs did not always keep pace with improvements in knowledge. Misconceptions about epidural safety, particularly fears of fetal exposure and long-term neurological harm, remained common even among women who received the intervention. These persistent concerns, despite corrective information, mirror findings from antenatal studies conducted in settings where medical mistrust and anecdotal stories strongly influence views of obstetric procedures (25). Thus, although knowledge scores improved, belief change on emotionally charged or risk-sensitive topics was more limited, suggesting that information alone may not overturn entrenched, culturally reinforced narratives (26).

The intervention’s design likely contributed to this gap. While the TTM supports tailoring content to different stages of behavior change, it does not directly target the emotional processes that shape health decisions. Without components that deliberately address affect, such as narrative reframing, guided dialog, or structured myth-challenging exercises, gains may remain primarily informational. Prior work on prenatal counseling emphasizes the inclusion of reflective elements when discussing topics characterized by uncertainty or procedural ambiguity (27). In their absence, women may filter new information through existing anxieties, selectively accepting what fits their prior beliefs and discounting what challenges them.

Regarding the childbirth experience, women in the intervention group did not report significantly different CEQ-C scores than those receiving routine education. Multivariable analyses showed a strong association between women’s global delivery-satisfaction ratings and CEQ-C scores; however, this finding should be interpreted cautiously because these variables are conceptually related, although assessed using separate measures. Occupational status was also associated with CEQ-C scores. This finding may reflect differences in expectations, perceived autonomy, or other unmeasured social and psychological factors; however, this interpretation is exploratory and should be confirmed in future studies (28–30). These results challenge the assumption that antenatal education alone reliably determines childbirth experience.

In this study, TTM-based antenatal education produced substantial improvements in KAP regarding neuraxial analgesia but did not yield statistically significant differences in overall childbirth experience, as measured by the CEQ-C. This finding is consistent with evidence that antenatal education primarily strengthens cognitive preparedness and self-efficacy, whereas women’s perceptions of childbirth are heavily shaped by intrapartum factors such as perceived control, quality of professional support, and opportunities to participate in decision-making (28, 31, 32). Feeling involved and supported during labor often has a greater impact on birth experience than information alone (30). Validation studies of the CEQ similarly highlight that its core domains (own capacity, professional support, perceived safety, and participation) are closely linked to communication and support during labor rather than to antenatal education in isolation (23, 33). Taken together with the present findings, this suggests that stage-tailored antenatal education should be paired with system-level efforts to improve intrapartum communication, team responsiveness, and workflow, so that the expectations and skills developed before birth can be reinforced in real time and translated into more positive childbirth experiences.

The implication is not that antenatal education is unimportant, but that its impact depends on how well it aligns with the broader care environment. Research in other areas of obstetric care indicates that systemic factors, such as communication style, continuity, and transparency, moderate the extent to which antenatal preparation leads to experiential benefits (34).

This misalignment suggests a need to rethink how antenatal education is conceptualized and delivered. Rather than functioning as a standalone module, it may be more effective as part of a longitudinal care pathway that includes postnatal feedback and iterative adjustment. Tailoring content to women’s informational and emotional needs, rather than solely to gestational age, could enable more nuanced programming. Findings from chronic disease management and surgical preparation show that aligning educational support with psychological readiness yields more durable behavior change and greater satisfaction (35). Clinically, this could involve standardizing core messages about labor analgesia, strengthening coordination between antenatal educators and intrapartum teams to ensure consistent information, and establishing structured follow-up mechanisms to support decision-making across pregnancy. Such an integrated approach would help ensure that gains achieved through antenatal preparation are sustained and effectively carried into intrapartum care.

### Limitations and future directions

This study has several limitations. First, we used a quasi-experimental design in which participants were assigned to the TTM-based intervention or the routine education group based on the parity of their enrollment sequence number, rather than through concealed randomization. Although baseline sociodemographic and obstetric characteristics were generally comparable, allocation based on enrollment order inherently increases the risk of selection bias because clinicians and participants could, at least in theory, anticipate group assignment. This lack of allocation concealment may have influenced which women agreed to participate at particular times or how actively they engaged with perinatal education resources, potentially favoring more motivated or health-conscious individuals in the intervention group and thereby overestimating the true effect of TTM-based education on KAP outcomes. Implementing fully randomized, concealed allocation was not feasible in this setting due to staffing constraints, clinic workflow, and the need to integrate the intervention into routine antenatal services; however, we sought to limit selection bias by applying consistent eligibility criteria, enrolling women consecutively, and comparing key baseline characteristics between groups.

Second, the sample was drawn from a single medical center and was of modest size, which may limit the generalizability of the findings. Although 200 women were initially allocated, the final analysis included 77 in the TTM group and 71 in the routine education group. The overall attrition rate was 26.0% (23.0% in the TTM group and 29.0% in the control group), mainly due to clinical events, including conversion to cesarean delivery and preterm birth. While baseline characteristics did not differ significantly between completers and excluded participants, analyses were conducted on a per-protocol basis rather than using an intention-to-treat approach, which may reduce the external validity of the results. Future studies should aim to minimize attrition and incorporate intention-to-treat analyses with appropriate imputation methods.

Third, no formal *a priori* sample-size or power calculation was performed, and the sample size was determined pragmatically according to the expected number of eligible participants and available resources. Consequently, the final sample may have been insufficient to detect modest between-group differences, particularly for secondary outcomes such as childbirth experience. In addition, women in the TTM group had more antenatal labor analgesia evaluations and online consultations than those in the routine education group, indicating higher levels of provider contact and supervision. It raises the possibility of engagement bias and differential intervention exposure, whereby more motivated women, or those receiving more intensive follow-up, might benefit more regardless of the TTM framework itself. As a result, the specific contribution of stage-based tailoring cannot be fully disentangled from the effect of increased contact time, and future randomized trials should carefully measure and, where possible, standardize intervention dose across groups.

Fourth, although the TTM emphasizes individualized intervention, participants’ stages of change were assessed through ongoing clinical judgment rather than a validated staging instrument (such as the URICA scale). Given the specialized nature of labor analgesia, most primiparous women at baseline were clustered in the Pre-contemplation or Contemplation stages as they were only beginning to consider pain management options. However, the lack of quantitative tracking of stage distribution and transitions limits our ability to provide a more fine-grained analysis of behavioral progression over time.

Fifth, multiple secondary outcomes were analyzed without formal adjustment for multiple comparisons, thereby increasing the risk of Type I error. These secondary findings should therefore be interpreted as exploratory and hypothesis-generating rather than confirmatory.

Sixth, the strong association between delivery satisfaction and CEQ-C score should be interpreted with caution. Delivery satisfaction was measured independently as a single global categorical item and was not included in the CEQ-C scoring algorithm. However, both variables capture related subjective aspects of childbirth and were assessed in the same postpartum context. Therefore, conceptual overlap and shared-method variance may have inflated the observed regression coefficient and contributed to the high model R^2^. The regression analysis should consequently be regarded as exploratory and descriptive rather than evidence that delivery satisfaction independently causes a more positive childbirth experience. Future studies should distinguish more clearly between global satisfaction and multidimensional childbirth-experience constructs, ideally using prospectively specified models and measures that minimize construct overlap.

Finally, in the study center, neuraxial labor analgesia is routinely offered to medically eligible primiparous women as part of standard care, and all participants ultimately received epidural analgesia. Consequently, the “practice” component of the KAP framework mainly captured preparatory and engagement behaviors (such as information seeking, attending antenatal evaluations and consultations, and planning for analgesia) rather than the decision to accept or decline neuraxial analgesia. Importantly, although the intervention increased preparatory and engagement behaviors around neuraxial analgesia, the choice to receive epidural analgesia remained voluntary, and women opting for non-pharmacological approaches were respected. Because the study was conducted in a setting where neuraxial analgesia is routinely offered as standard care and all analyzed participants ultimately received it, we could not directly assess how TTM-based education influences the decision to accept or decline neuraxial analgesia. It limits our ability to examine how KAP levels relate to differential uptake of epidural analgesia, and future research in settings with greater variability in analgesia use is needed to investigate this relationship more directly.

Future studies should therefore prioritize multicenter randomized controlled trials with larger and more diverse samples, standardized intervention dosing, validated staging measures, and extended follow-up to confirm and extend the generalizability of these findings.

## Conclusion

TTM-based health education was more effective than routine education in improving pregnant women’s knowledge, attitudes, and practices related to painless delivery. Building on these findings, integrating stage-tailored educational strategies into routine antenatal care may help optimize maternal preparation and support evidence-based decision-making during childbirth.

## Data Availability

The original contributions presented in the study are included in the article/Supplementary material, further inquiries can be directed to the corresponding author.
