# Supplementary material for: Effect of health education based on the Transtheoretical Model on pregnant women’s knowledge, attitudes, and practices toward painless delivery: a quasi-experimental study
Source: Front Public Health. 2026 Sep 17;14:1763939. doi: 10.3389/fpubh.2026.1763939 (PMC13599620; doi:10.3389/fpubh.2026.1763939)
Supplement: Supplementary file 1 [file Table_1.docx]

**Supplementary Table S1.** TTM-Based Labor Analgesia Health Education Strategy for Primiparas

| **Stage** | **Characteristics** | **Goals** | **Intervention Strategies** |
| --- | --- | --- | --- |
| **Pre-contemplation** | 1. Has no prior knowledge of labor analgesia.  2. Is unaware of what methods the doctor will use to manage pain.  3. Is uncertain whether to opt for labor analgesia. | Establish a basic understanding of neuraxial labor analgesia among pregnant women. | 1. Assess each patient's general status, prenatal visit history, and psychological well-being; evaluate their labor analgesia knowledge, attitudes, and practices (KAP); administer the Self-Rating Anxiety Scale (SAS) and Self-Rating Depression Scale (SDS).  2. Educate patients on what neuraxial labor analgesia is, why it is the preferred approach, its benefits and limitations, the level of risk involved, and strategies for avoiding complications, providing an accessible foundation for informed decision-making.  3. Address patient questions and begin building a trusting therapeutic relationship. |
| **Contemplation** | 1. Has a basic understanding of labor analgesia.  2. Remains skeptical about the safety and efficacy of labor analgesia; unclear on how to actively participate.  3. Open to trying it if deemed necessary. | Build confidence in labor analgesia among pregnant patients. | 1. An anesthesiologist conducts an initial screening of each enrolled patient for contraindications to neuraxial labor analgesia.  2. Establish a dedicated messaging group and regularly distribute curated labor analgesia content and short educational videos.  3. Key topics, including physical and psychological preparation, common concerns, and the complete labor process, are broken into focused online modules and delivered as structured educational sessions.   1) The implementation procedure, timing of initiation, patient cooperation and experience, onset time, analgesic efficacy, and changes in labor progression, covering the entire delivery process. 2) Why is neuraxial anesthesia the preferred method for labor analgesia. 3) When labor analgesia is initiated, and whether pain persists after it takes effect. 4) Can labor analgesia be administered if I have a recent cold or fever? 5) Is labor analgesia safe if I have pre-existing back pain, and could it worsen symptoms? 6) Is labor analgesia an option if I have elevated blood pressure or blood glucose? 7) Under what circumstances is labor analgesia contraindicated? 8) How can I improve fetal hypoxia tolerance and increase the likelihood of vaginal delivery? 9) What diet and exercise strategies during pregnancy can support natural childbirth? 10) What preparations should I make before going into labor?   4. Invite recently delivered multiparous women to share first-hand experiences with labor analgesia; facilitate in-depth peer dialogue to help reduce prenatal anxiety.  5. Guide patients through structured self-assessment covering prenatal health status, comorbid conditions, psychological well-being, exercise habits, and lifestyle; engage in thorough discussions of current concerns and outstanding questions about labor analgesia, with all queries managed and resolved collaboratively by the labor analgesia education team. |
| **Preparation** | 1. Has a solid foundational understanding of labor analgesia  2. Based on individual patient needs, develop a tailored plan covering prenatal check-ups, physical activity, and nutrition. | Develop an individualized labor analgesia plan. | 1. Conduct a thorough assessment of the patient's health status and establish a dedicated follow-up group. Evaluate for scoliosis, back injuries, lumbar disc herniation, prior lumbar or leg surgery, pain in the lumbar, gluteal, leg, or inguinal regions, back skin infections, abnormal hemoglobin, platelet count, or coagulation function, and blood pressure/glucose control. Based on findings, develop a personalized treatment and preparation plan addressing relevant issues, such as skin infection treatment, anemia management, weight control, and blood pressure/glucose optimization, to ensure readiness for labor analgesia.  2. Qualified obstetricians and obstetric nurses provide detailed guidance on prenatal dietary guidelines, exercise recommendations, and general prenatal health. This reinforces awareness of pre-existing health conditions and unhealthy lifestyle habits; patients receive individualized health education prescriptions and begin developing concrete prenatal health management goals.  3. Provide targeted education to family members and support persons. Encourage them to learn about labor analgesia and dispel common misconceptions about neuraxial anesthesia, such as beliefs that it increases postpartum back pain or causes cognitive impairment. Engage family members as active partners in supporting adherence to the health plan. |
| **Action and maintenance** | 1. Supervise and support completion of the prenatal health plan; ensure physical and psychological readiness for labor analgesia.  2. Implement a personalized one-on-one follow-up tracking mechanism via phone, online communication, and other channels. | Promote sustained positive behavior change and reinforce adherence. | 1. Encourage active engagement in behavior change: pursue treatment for anemia and coagulation abnormalities, engage in appropriate prenatal exercise, maintain a healthy weight, stabilize blood pressure and blood glucose, and take precautions against upper respiratory infections and accidental injury.  2. Remind patients to attend all scheduled prenatal check-ups; adjust exercise and dietary plans based on clinical findings and laboratory results. Initiate pharmacological treatment promptly and appropriately when indicated.  3. Hold weekly online labor analgesia classes; encourage both patients and their families to participate. Sessions aim to deepen understanding of the full labor analgesia process, strengthen self-management skills, build patient confidence, and support physical and psychological preparation ahead of delivery.  4. Team members engage actively with patients in the group to monitor health plan adherence and emotional well-being. Patients are encouraged to share experiences and support one another. Questions and errors are addressed promptly, with practical guidance provided as needed.  5. Maintain consistent follow-up to reinforce positive behaviors and sustain confidence in labor analgesia through to delivery. |
