# Supplementary material for: Effect of health education based on the Transtheoretical Model on pregnant women’s knowledge, attitudes, and practices toward painless delivery: a quasi-experimental study
Source: Front Public Health. 2026 Sep 17;14:1763939. doi: 10.3389/fpubh.2026.1763939 (PMC13599620; doi:10.3389/fpubh.2026.1763939)
Supplement: Supplementary file 2 [file Table_2.docx]

**Supplementary Table S2. Reasons for exclusion by study group**

| **Reason for exclusion** | **TTM group (n = 23)** | **Control group (n = 29)** | **Total (n = 52)** |
| --- | --- | --- | --- |
| Cesarean section (medical indication) | 10 (43.5) | 11 (37.9) | 21 (40.4) |
| Cesarean section (social/elective) | 4 (17.4) | 9 (31.0) | 13 (25.0) |
| Emergency cesarean after attempted vaginal delivery | 5 (21.7) | 6 (20.7) | 11 (21.2) |
| Preterm delivery (< 37 weeks) | 1 (4.3) | 2 (6.9) | 3 (5.8) |
| Did not receive neuraxial analgesia | 1 (4.3) | 1 (3.4) | 2 (3.8) |
| Incomplete data / lost to follow-up | 2 (8.7) | 0 (0.0) | 2 (3.8) |
