# Supplementary material for: Effect of health education based on the Transtheoretical Model on pregnant women’s knowledge, attitudes, and practices toward painless delivery: a quasi-experimental study
Source: Front Public Health. 2026 Sep 17;14:1763939. doi: 10.3389/fpubh.2026.1763939 (PMC13599620; doi:10.3389/fpubh.2026.1763939)
Supplement: Supplementary file 3 [file Table_3.docx]

**Supplementary Table S3. Comparison of baseline characteristics between study completers and excluded participants**

| **Variables** | **Completed the study (n = 148)** | **Excluded (n = 52)** | **Statistic** | **P** |
| --- | --- | --- | --- | --- |
| **Age (years)** | 29 (28–32) | 30 (27–33) | U = 3628.5 | 0.540 |
| **Group, n (%)** |  |  | χ² = 0.650 | 0.420 |
| TTM group | 77 (52.0) | 23 (44.2) |  |  |
| Control group | 71 (48.0) | 29 (55.8) |  |  |
| **Residence, n (%)** |  |  | χ² = 0.254 | 0.614 |
| Urban | 107 (72.3) | 35 (67.3) |  |  |
| Rural/Suburban | 41 (27.7) | 17 (32.7) |  |  |
| **Education, n (%)** |  |  | χ² = 0.480 | 0.923 |
| Senior high school and below | 18 (12.2) | 5 (9.6) |  |  |
| College | 37 (25.0) | 12 (23.1) |  |  |
| Bachelor’s degree | 64 (43.2) | 25 (48.1) |  |  |
| Master’s degree and above | 29 (19.6) | 10 (19.2) |  |  |
| **Occupation, n (%)** |  |  | χ² = 1.042 | 0.594 |
| Professional/managerial | 44 (29.7) | 17 (32.7) |  |  |
| General staff | 63 (42.6) | 18 (34.6) |  |  |
| Service/other | 41 (27.7) | 17 (32.7) |  |  |
| **Monthly income (CNY), n (%)** |  |  | χ² = 6.238 | 0.101† |
| 2000-5000 | 34 (23.0) | 12 (24.0) |  |  |
| 5000-10,000 | 59 (39.9) | 21 (42.0) |  |  |
| 10,000-20,000 | 39 (26.4) | 17 (34.0) |  |  |
| > 20,000 | 16 (10.8) | 0 (0.0) |  |  |
| **Insurance type, n (%)** |  |  | χ² = 6.519 | 0.089† |
| Social insurance only | 75 (50.7) | 22 (42.3) |  |  |
| Commercial insurance only | 0 (0.0) | 2 (3.8) |  |  |
| Both | 52 (35.1) | 19 (36.5) |  |  |
| None | 21 (14.2) | 9 (17.3) |  |  |

*† Some expected cell counts < 5; results should be interpreted with caution.*

*Continuous variables compared using the Mann–Whitney U test; categorical variables compared*
